# At the Crossroads of Health and Disease: Consequences of Fat in the Liver

**DOI:** 10.1146/annurev-physiol-022724-105515

**Published:** 2025-02

**Authors:** Matthew Dukewich, Liyun Yuan, Norah A. Terrault

**Affiliations:** Division of Gastrointestinal and Liver Diseases, University of Southern California, Los Angeles, California, USA

**Keywords:** metabolic dysfunction–associated steatotic liver disease, MASLD, alcohol-associated liver disease, ALD, lipogenesis, lipotoxicity, metabolic health

## Abstract

The liver plays a central role in regulating lipid and glucose metabolism, particularly in transitioning between energy storage and provision in fed and fasting states. Loss of metabolic flexibility, characterized by the impaired capacity to shift between different energy substrates, sets the stage for accumulation of hepatic triglyceride as lipid droplets and further metabolic perturbations. Cross talk between the liver and other organs, including adipose tissue, pancreas, and muscle, is relevant in this transition. In addition to the metabolic consequences of steatosis, there are significant liver risks related to triggered inflammatory and fibrotic processes. Steatotic liver diseases affect an estimated one in three adults globally and contribute to substantial morbidity and mortality. This review focuses on the liver’s role in lipid metabolism, defining metabolic health and unhealth, the pathogenic underpinnings that lead to steatohepatitis and hepatic fibrosis, and the clinical features and therapies for the most common forms of steatotic liver diseases.

## 1. INTRODUCTION

Hepatic steatosis is defined by the accumulation of fat within hepatocytes, primarily as triglyceride (TG), in greater than 5% of hepatocytes and is remarkably common. Population-based estimates from the United States, Europe, and Asia suggest that one in three adults has hepatic steatosis (1). Recently changed from fatty liver to steatotic liver disease (SLD), this umbrella term includes metabolic dysfunction–associated steatotic liver disease (MASLD), formerly known as nonalcoholic fatty liver disease, alcohol-associated liver disease (ALD), an overlap group termed MetALD, and other secondary causes (e.g., mediation associated, viral associated) (2) (Table 1). MASLD is the leading cause of steatotic liver disease, affecting approximately one quarter of the global population. A significant portion of individuals with hepatic steatosis will develop liver inflammation (steatohepatitis) and fibrosis and be at risk for cirrhosis and liver cancer. The tipping point at which liver fat disrupts liver health and overall health is multifaceted and complex. This review focuses on the liver’s role in lipid metabolism, defining metabolic health and unhealth, the pathogenic underpinnings that lead to steatohepatitis and hepatic fibrosis, and the clinical features and therapies for specific steatotic liver diseases.

## 2. LIVER AND NORMAL METABOLISM

**Figure 1:**
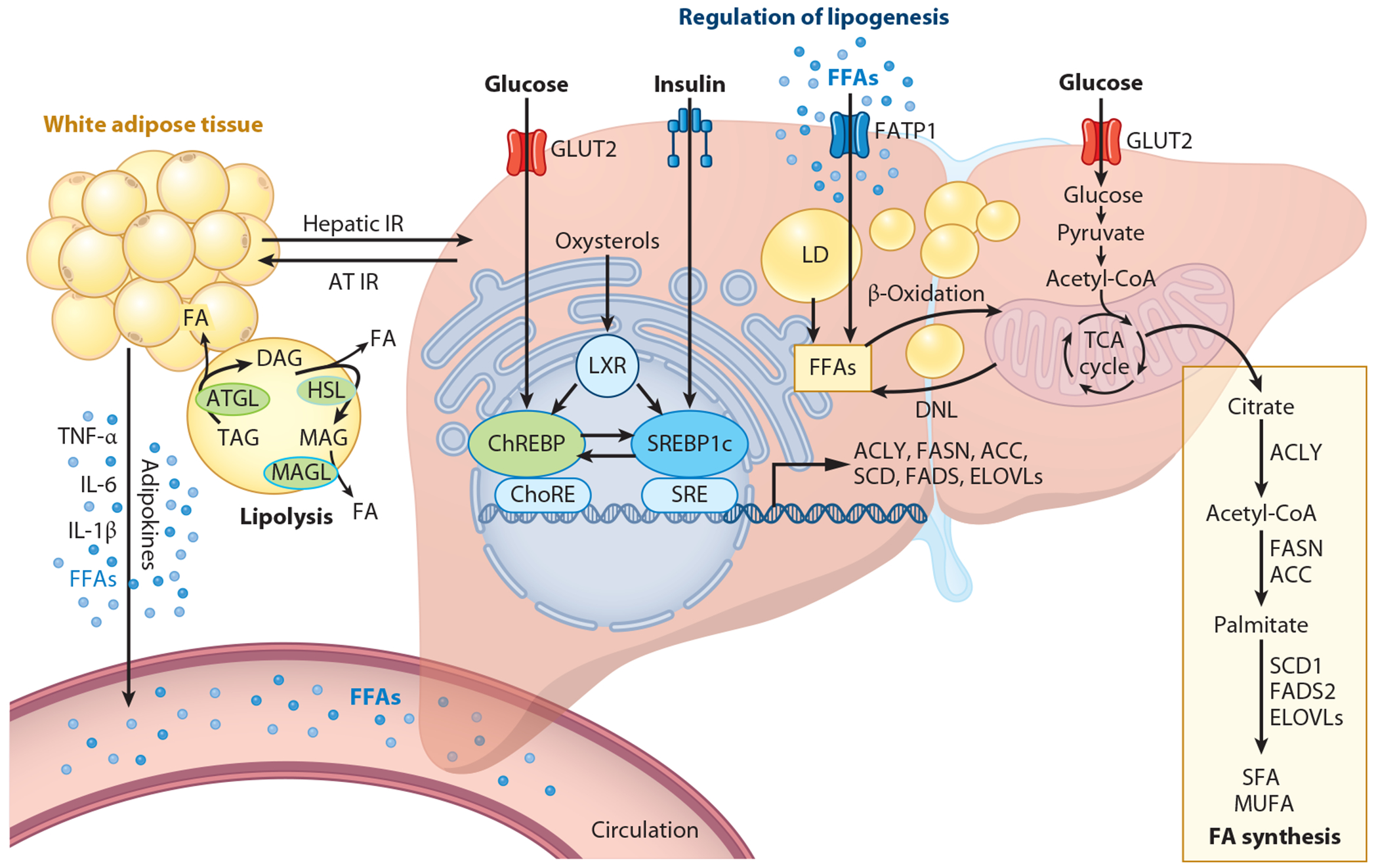
Regulation of hepatic lipogenesis. The synthesis of TAG involves the activation of FFAs into acyl-CoA by the enzyme acyl-CoA synthetase. The pool of FFAs within hepatocytes originates primarily from three sources: DNL, influx from dietary intake and adipose tissue lipolysis, and hydrolysis from TAG stored in LDs. During the fed state, glucose is taken up by insulin-regulated GLUT2 into the cytosol, where it undergoes glycolysis, converting into pyruvate. Pyruvate is then transported into mitochondria for further oxidation in the TCA cycle. Citrate, an intermediate of the TCA cycle, is subsequently exported into the cytosol, where it serves as a substrate for DNL. Lipogenesis regulation primarily occurs at the transcriptional level, controlled by ChREBP and SREBP1c. These transcription factors promote the expression of key genes involved in FA synthesis, including ACLY, ACC1, FASN, and SCD1. Insulin-regulated GLUT2, FATP, and IR are integral to this regulatory process. Abbreviations: ACC1, acetyl-CoA carboxylase 1; ACLY, ATP-citrate lyase; AT, adipose tissue; ATGL, adipose triglyceride lipase; ChoRE, carbohydrate-response element; ChREBP, carbohydrate response element-binding protein; DAG, diacylglycerol; DNL, de novo lipogenesis; ELOVL, elongation of very-long-chain fatty acid protein; ER, endoplasmic reticulum; FA, fatty acid; FADS2, fatty acid desaturase 2; FASN, fatty acid synthase; FATP1, fatty acid transport protein-1; FFA, free fatty acid; GLUT2, glucose transporter type 2; HSL, hormone-sensitive lipase; IL-6, interleukin 6; IL-1β, interleukin 1β; IR, insulin receptor; LD, lipid droplet; LXR, liver X receptor; MAG, monoacylglycerol; MAGL, monoacylglycerol lipase; MUFA, monounsaturated fatty acid; SCD, stearoyl-CoA desaturase; SCD1, stearoyl-CoA desaturase-1; SFA, saturated fatty acid; SREBP1c, sterol regulatory element-binding protein 1c; TAG, triacylglycerol; TCA, tricarboxylic acid; TNF-α, tumor necrosis factor-alpha. Figure adapted from image created in BioRender; Yuan L. 2024. https://BioRender.com/c52r025.

The liver plays a central role in regulating lipid and glucose metabolism. Hepatocytes possess remarkable metabolic flexibility in transitioning between tasks of energy storage and provision during fed and fasted states, respectively. During the fasting state, insulin levels are low, and lipolysis is stimulated, leading to the release of fatty acids (FAs) from white adipose tissue to provide energy for various tissues. Concurrently, the liver upregulates gluconeogenesis and glucose secretion, supplying glucose to glucose-dependent cells and tissues. Skeletal muscle relies primarily on FAs for fuel rather than glucose. In the fed state, increased insulin leads to the inhibition of lipolysis in adipose tissue and promotes glucose uptake by the liver and muscle. In the liver, de novo FA synthesis occurs within hepatocytes from nonlipid precursors, primarily carbohydrates, a process referred to as de novo lipogenesis (DNL). DNL in the liver is regulated by insulin, glucose, and a complex interplay of enzymatic and nuclear transcription factor pathways [including farnesoid X receptor (FXR) and peroxisome proliferator–activated receptors (PPARs)]. Newly synthesized FAs can be directed toward the endoplasmic reticulum (ER) for esterification, predominantly forming TGs, and either secreted into the systemic circulation within very-low-density lipoproteins (VLDLs) or stored as lipid droplets (LDs) within the cytosol. Hepatic FA clearance also occurs via FA oxidation pathways (Figure 1).

The regulation of lipogenesis is intricately controlled in response to various nutrients and metabolic hormones, including insulin, glucagon, and glucocorticoids. Key regulators such as sterol regulatory element-binding proteins (SREBPs), carbohydrate response element-binding protein (ChREBP), and liver X receptor alpha (LXR-α) play essential roles in this process. SREBPs, located in the ER membrane, undergo post-translational processing to transition into the nucleus, where they stimulate the transcription of essential genes for FA synthesis. Upon insulin stimulation, the precursor form of SREBP is escorted by the SREBP cleavage-activating protein from the ER membrane to the Golgi complex, where it undergoes cleavage by proteolytic enzymes [sphingosine 1-phosphate and sphingosine 2-phosphate (S1P and S2P)] (3). The mature forms of SREBPs, SREBP1c, and SREBP2 then relocate into the nucleus to initiate the expression of key lipogenic genes. SREBP2 also plays a vital role in maintaining cholesterol homeostasis. ChREBP promotes lipogenesis in response to glucose stimulation by forming a heterodimeric complex with Max-like protein X, which binds to a carbohydrate-response element. ChREBP has two isoforms, ChREBP-α and ChREBP-β, whose expression is modulated by metabolic signals such as feeding, glucose levels, and obesity. There is a synergistic relationship between SREBP1c and ChREBP. SREBP1c ablation reduces the expression of ChREBP messenger RNA (mRNA) levels, while deletion of ChREBP results in decreased mRNA and protein levels of SREBP1c (4). The overlapping yet distinct roles of SREBP1c and ChREBP enable cells to synthesize lipids primarily in the presence of both insulin and carbohydrates. In the absence of ChREBP, SREBP1c overexpression restores the expression of lipogenic genes but not glycolytic genes. Conversely, in the SREBP cleavage-activating protein knockout model suppressing SREBP, ChREBP reinstates the glycolytic gene program without fully rescuing lipogenic gene expression. Thus, both SREBP1c and ChREBP are necessary for the coordinated induction of glycolytic and lipogenic gene expression to ensure proper lipogenic activation (4). LXR-α, a nuclear hormone receptor activated by oxysterols and insulin, also plays a role in the expression of lipogenic genes. It forms heterodimers with the retinoid X receptor and binds to LXR elements in the promoters of its target genes in the presence of oxysterols (5). LXR-α directly stimulates lipogenic gene expression by binding to target promoters and indirectly by enhancing SREBP1c transcription. Under normal healthy conditions, DNL serves as a protective mechanism against lipotoxicity.

TG and cholesterols are stored in LDs. TGs and cholesterol esters form the neutral core of LDs, which are enveloped by a phospholipid monolayer consisting of multiple LD proteins trafficking from the ER bilayer to the LD membrane (class I LD proteins) or remaining associated with cytosolic LDs (class II LD proteins). Nascent LDs bud from the ER outer membrane with the coordinated action of several key enzymes such as diacylglycerol *O*-acyltransferase 1 (DGAT1), DGAT2, acetyl-CoA acetyltransferase 1 (ACAT1), and ACAT2. DGAT1 facilitates the synthesis of TG from DAG, while DGAT2 facilitates TG synthesis from FAs. ACAT1 converts cholesterol into cholesteryl esters and ACAT2 releases the product in lipoproteins, preventing cytotoxicity from cholesterol crystallization. Seipin assembly protein complex forms a cage-like structure, organizing lipid composition at ER sites and priming LD growth and budding (6). One well-validated genetic polymorphism linked to SLD is PNPLA3 (Table 2). PNPLA3 encodes a class I ER bilayer LD protein, which carries TG hydrolase activity. The substitution of isoleucine with methionine at position 148 (I148M) of PNPLA3 results in a missense variant (PNPLA3 I148M), which exhibits reduced hydrolase activity. As a result, the accumulation of large LDs is observed in affected individuals (7). Overexpressing PNPLA3 (I148M) in the liver of transgenic mice reinstates the fatty liver phenotype and insulin resistance (8). This modification impacts TG metabolism within LDs by increasing the synthesis of FAs and TG, hindering TG hydrolysis, and reducing TG long-chain polyunsaturated FAs (PUFAs) (8).

## 3. LIVER FAT AND UNHEALTHY METABOLISM

A key feature of an unhealthy metabolism is a loss of metabolic flexibility, characterized by impaired capacity to shift between different energy substrates such as free fatty acids (FFAs) and glucose. In scenarios of chronic nutritional surplus and caloric overload, adipocytes in the subcutaneous adipose tissue eventually reach their maximum storage capacity for lipid. Consequently, excess FFAs are redirected to visceral adipose and ectopic sites, including the liver, muscle, and pancreas, where they contribute to insulin resistance. Insulin resistance is defined as impaired insulin-mediated effects in several organs, including impaired glucose uptake in skeletal muscle, impaired suppression of glucose production in the liver, and impaired suppression of lipolysis in adipose tissue. There is a direct relationship between tissue (hepatic, skeletal muscle, and adipose) insulin resistance and hepatic fat content, independent of body mass index (BMI), percentage of body fat, and visceral organ fat (9–13). Adipose tissue insulin resistance increases the flux of FFAs to the liver, inducing hepatic insulin resistance and enhancing glucose production, de novo hepatic lipogenesis, VLDL release, and atherogenic dyslipidemia. Persistent postprandial hyperinsulinemia and increased circulating FFA levels suppress glucose release and promote lipid synthesis from carbohydrates within the liver. Increased FA deposition in muscle promotes insulin resistance, inhibiting insulin-mediated glucose uptake into muscle and further exacerbating the glucose delivered to the liver and fueling more DNL. FFAs spill over into the pancreas, causing β-cell dysfunction by lipotoxicity, hyperglycemia, and diabetes. Increased liver fat also promotes hepatic glucagon resistance.

Any amount of intrahepatic TG accumulation increases risk for insulin resistance, without a threshold effect (10, 14). The relationship between obesity and insulin resistance is driven by adipose tissue inflammation, which stimulates cytokine and chemokine release [including interleukin 6 (IL-6), tumor necrosis factor-alpha (TNF-α), and chemokine (C-C motif) ligand 2 (CCL2)] and macrophage recruitment (9). Adipose tissue inflammation occurs with the phenotypic switch of adipose tissue to an inflammatory subtype associated with immune cell infiltration. Excess calorie intake and increased adipose tissue accumulation appear to trigger this inflammatory switch (15). Adipose tissue also produces peptide hormones that are associated with insulin signaling pathways, including resistin, retinol-binding protein 4, adiponectin, and leptin. Adiponectin is the most abundant of these secreted proteins, and plasma concentrations are inversely associated with presence of hepatic steatosis, insulin resistance, type 2 diabetes mellitus (T2DM), and metabolic syndrome (9).

In addition to these adipokine effects, obesity is associated with systemic lipolysis and release of FFAs. The rate of FFA release into the circulation is directly proportional to fat mass. Overall, alterations in adipose tissue lipolytic activity, hepatic lipolysis of circulating TGs, and tissue FFA transport proteins are involved in the pathogenesis of hepatic steatosis (9). Elevated circulating FFAs lead to intrahepatic TG accumulation that subsequently worsens intrahepatic insulin resistance. Obesity is associated with genetic upregulation of hepatic lipase and lipoprotein lipase, providing a link between excess peripheral adipose tissue and increased incorporation of FAs as hepatic steatosis. Intracellular intermediates of FA metabolism are believed to interfere with insulin activity through activation of inflammatory pathways, particularly via activation of protein kinase C and mammalian target of rapamycin, inhibition of Akt, and activation of nuclear factor kinase B (NF-κB). Experimental models suggest that the pathways that mediate insulin resistance exhibit tissue-specific differences. Increased intrahepatic NF-κB is associated with obesity, hepatic steatosis, insulin resistance, and plasma levels of IL-6. Hepatocellular NF-κB may lead to intrahepatic insulin resistance independent of steatosis (9). Several models demonstrate hepatic TG accumulation without insulin resistance, such as overexpression of hepatic DGAT, blockade or decrease of VLDL secretion (hypobetalipoproteinemia), pharmacologic blockage of FA oxidation, and genetic apoB synthesis deficiency in humans (9).

## 4. HEPATIC FAT AND ASSOCIATED INFLAMMATION AND FIBROSIS

**Figure 2:**
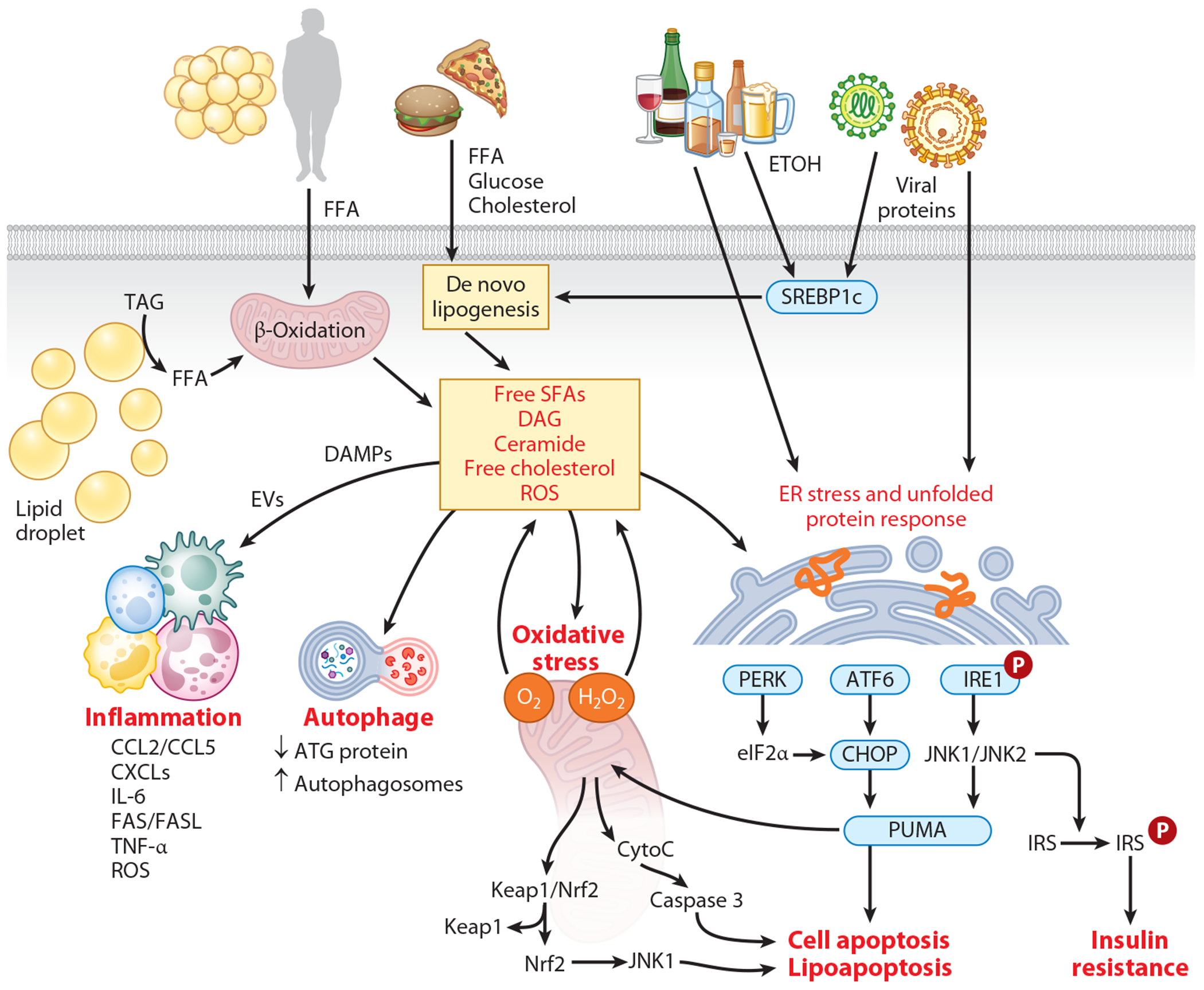
Mechanisms underlying lipid-related hepatocyte injury. Obesity and insulin resistance commonly disrupt de novo lipogenesis and disturb the balance of lipid influx and efflux. Consequently, toxic lipid species such as free SFAs, DAG, and cholesterol accumulate, leading to lipotoxicity. Lipotoxicity initiates the generation of ROS, induces ER stress, impairs autophagy, and activates inflammatory responses. These processes further trigger apoptotic signaling pathways involving proteins such as JNK and Bcl-2 or necroptotic signaling pathways. Viral infections such as HCV and HIV, as well as excessive alcohol consumption, also disrupt lipid metabolism, leading to hepatic steatosis and liver injury. Abbreviations: ATF6, activating transcription factor 6; ATG, autophagy-related gene; Bcl-2, B-cell lymphoma 2; CCL2/5, chemokine (C-C motif) ligand 2/5; CHOP, C/EBP homologous protein; CXCLs, chemokine (C-X-C motif) ligands; DAG, diacylglycerol; DAMPs, damage-associated molecular patterns; eIF2α, eukaryotic initiation factor 2-alpha; ER, endoplasmic reticulum; ETOH, ethanol (alcohol); EVs, extracellular vesicles; FAS/FASL, Fas cell surface death receptor/Fas ligand; FFA, free fatty acid; HCV, hepatitis C virus; IL-6, interleukin 6; IRE1, inositol-requiring enzyme 1; IRS, insulin receptor substrate; JNK1/2, c-Jun N-terminal kinase 1/2; Keap1, Kelch-like ECH-associated protein 1; Nrf2, nuclear factor erythroid 2-related factor 2; PERK, protein kinase R-like endoplasmic reticulum kinase; PUMA, p53 upregulated modulator of apoptosis; ROS, reactive oxygen species; SFA, saturated fatty acid; SREBP1c, sterol regulatory element-binding protein 1c; TAG, triacylglycerol; TNF-α, tumor necrosis factor-alpha. Figure adapted from image created in BioRender; Yuan L. 2024. https://BioRender.com/f89m453.

The pathobiology of steatohepatitis is related to the accumulation of FAs and other lipotoxic species in the liver (Figure 2). As the hepatic FAs accumulate, mitochondria initially acclimate by increasing their sizes, rates of biogenesis, and oxidative capacities. However, these compensatory mechanisms become insufficient with the overload of FAs condensed into TGs, contributing to hepatic steatosis and the increased oxidation in microsomes and peroxisomes and causing oxidant stress and cellular damage. The by-products, reactive oxygen species (ROS), are considered a major source of liver injury, not only as signaling mediators but also by causing damage to DNA, proteins, and lipids. Oxidized lipids and phospholipids promote oxidative stress and inflammation. Additionally, ROS production may impair normal mitochondria turnover and impede fission/fusion cycles, resulting in arrested mitophagy, accumulation of damaged mitochondria, and induction of the unfolded protein response (UPR) (16).

Lipotoxicity is central to hepatocyte injury and inflammation as well as stellate cell activation leading to fibrosis. Saturated FAs like palmitate and stearate, found abundantly in the diet or synthesized de novo from carbohydrates, exert potent toxic effects, inducing apoptosis and inflammation through various mechanisms (17, 18). Monounsaturated FAs, such as oleate, primarily depend on the activity of stearoyl-CoA desaturase and are less toxic than palmitate acids. On the one hand, they may help mitigate cell death by reducing proapoptotic protein levels and sequestering palmitate acid in TGs. PUFAs, on the other hand, exert hepatocyte-protective effects. The n-6 PUFA alpha-linolenic acid has been shown to reduce apoptosis and suppress proinflammatory signaling (19). Ceramides are synthesized in the ER by the condensation of palmitoyl CoA (or another FA moiety) with sphingosine and can modulate the expression of Srebf1. Ceramide can alter the subcellular distribution of CD36, a protein involved in the uptake of FAs, thereby enhancing the uptake of FAs into hepatocytes, and can block the phosphorylation of AKT/PKB, a key signaling molecule involved in insulin-mediated suppression of gluconeogenesis. These actions of ceramides collectively contribute to increased fat deposition and glucose output, resulting in a state of selective insulin resistance (20). Ceramide and its derivative S1P have been implicated in apoptosis triggered by death ligands such as TNF-α and Fas ligand (21). Increased mitochondrial free cholesterol impairs the function of the 2-oxoglutarate carrier, which in turn reduces mitochondrial glutathione levels, promoting mitochondrial ROS generation, lipid peroxidation, and hepatocyte necrosis and apoptosis (22).

Various factors such as glucose-induced insulin secretion, nutrient overload, increased membrane cholesterol content, oxidative stress, and hypoxia can disrupt ER homeostasis, resulting in ER stress. In response to ER stress, the UPR is activated, which involves key players such as PERK, IRE1α, and ATF6α. Under lipotoxic conditions, both IRE1α and PERK can become activated. Once activated, IRE1α promotes the transcription of serine palmitoyltransferase genes via XBP1s, leading to ceramide biosynthesis and the subsequent release of extracellular vesicles from hepatocytes. These extracellular vesicles then recruit macrophages to the liver, promoting inflammation and injury. ER stress also induces caspase-2 expression, which colocalizes with and cleaves S1P. This cleavage releases active S1P and activates SREBP1 and SREBP2, promoting FA and cholesterol synthesis (23). Cholesterol accumulation in the ER membrane impairs sarco-/endoplasmic reticulum Ca^2+^-ATPase (SERCA) activity, reducing the ER’s protein-folding capacity and exacerbating UPR and ER stress (24). Additionally, some studies suggest that accumulation of free cholesterol may directly activate hepatic stellate cells, possibly through a Toll-like receptor 4-dependent pathway (25).

Different programmed cell death mechanisms including autophagy, apoptosis, ferroptosis, and pyroptosis have been implicated in the pathogenesis of MASLD and ALD. Both necroptosis and apoptosis were observed as major forms of cell death of hepatocytes when exposed to palmitate acids. Ferroptosis is marked by the iron-dependent buildup of lipid peroxides (oxidatively damaged phospholipids) and is associated with MASLD and ALD (26). Pyroptosis is a type of cell death linked to inflammasome activation, specifically NLRP3-mediated caspase-1 activation. This leads to pore formation in the cell membrane and eventual cell rupture. It is hypothesized that cholesterol crystallization may trigger the activation of the NLRP3 inflammasome in Kupffer cells (24).

Apoptosis is characterized with chromatin condensation and shrinkage of cells, while necroptosis is a regulated necrotic form of cell death characterized with cell swelling and subsequent rupture followed by membrane pore formation. The c-Jun N-terminal kinase (JNK) pathway serves as a key mediator for apoptotic signaling. Within hepatocytes, two JNK genes, *JNK1* and *JNK2*, are present. *JNK1* is particularly important in inducing apoptosis by upregulating the expression of proapoptotic BH3-only proteins from the Bcl-2 pathway, such as PUMA, BIM, and Bax (18, 27, 28). Upon activation of PUMA, Bax is activated and initiates the release of cytochrome C from the mitochondria, which in turn activates caspases and induces apoptosis. *JNK1*-dependent PUMA expression contributes to hepatocyte lipoapoptosis. Administration of a nonselective JNK inhibitor, SP600125, to the murine hepatocyte cell line has been demonstrated to reduce PUMA expression and decrease lipoapoptosis (27). JNK also mediates apoptosis by upregulation of TRAIL receptor 2 (TNFRSF10B) (29, 30). The interaction of JNK with mitochondrial Sab (SH3BP5) on the membrane of mitochondria leads to the generation of ROS and apoptosis (31). Upstream kinase, apoptosis signal-regulating kinase 1 (ASK1), is critical in regulating JNK activation. ASK1 is upregulated in the liver of individuals with steatohepatitis. The deubiquitinase, TNF-α-induced protein 3, which directly interacts with and deubiquitinates ASK1 in hepatocytes, ameliorates the onset and progression of metabolic dysfunction-associated steatohepatitis (MASH) (32). Additionally, CASP8 and FADD-like apoptosis regulator directly inhibit ASK1 by blocking its N terminus–mediated dimerization, leading to attenuation of the progression of steatohepatitis and metabolic disorders in mice and monkeys (33).

Necroptosis has been implicated as a major form of hepatocyte injury in SLD. The fragments/debris of cells as the result of necroptosis release danger-associated molecular patterns (DAMPs) and pathogen-associated molecular patterns (PAMPs), which initiate and propagate the proinflammatory process in the recruitment of macrophages and Kupffer cells. Necroptosis involves the formation of the necrosome, a protein kinase complex composed of receptor-interacting serine/threonine-protein kinase 1 (RIPK1), RIPK3, and the pseudokinase mixed-lineage kinase domain-like (MLKL) protein 1 (34). Elevated levels of RIP3 and MLKL were observed in the liver tissue and serum among patients with MASH (35, 36). Inhibiting either RIPK1 or RIPK3 improves steatohepatitis features in high fat diet (HFD)-fed mice and reverses steatosis in the methionine-choline-deficient diet model of MASLD. Inhibition of RIP1 with necrostatin-1 or small interfering RNA knockdown of RIP3 reduced palmitic acid–induced cytotoxicity (37).

Autophagy may protect against steatosis and progression to steatohepatitis by limiting hepatocyte injury. Downregulation of autophagy is observed in MASH, obesity, and alcohol-associated liver injury (38, 39). Soluble FAs, including omega-3 and omega-6 PUFAs, have been shown to promote autophagy and M2-mediated macrophage polarization, while also reducing ER stress in murine liver and adipose tissue (40, 41). The mechanisms by which autophagy protects against liver injury in the setting of hepatic steatosis include (*a*) removal of damaged organelles, such as mitochondria (source of ROS), or proteins that causes cellular dysfunction (42); (*b*) inhibition of hepatocyte death receptor-mediated apoptosis from TNF and Fas (43); and (*c*) cross talk with apoptotic pathways (44, 45).

## 5. METABOLIC DYSFUNCTION–ASSOCIATED STEATOTIC LIVER DISEASE

MASLD is defined by hepatic steatosis in conjunction with at least one cardiometabolic risk factor and low or no alcohol use (Table 1); it represents a continuum from simple steatosis to steatohepatitis with or without fibrosis to advanced fibrosis and cirrhosis. MASH, the inflammatory form of steatotic liver disease, is associated with progression to fibrosis and cirrhosis (46). Histological classification of MASH is based on a semiquantitative scoring system including histologic features of fibrosis (stages F0–F4), steatosis, lobular inflammation, and hepatocellular ballooning. Inflammation is generally located in a perisinusoidal distribution in early stages of disease and progresses to a panlobular distribution with disease progression (47). The presence of active steatohepatitis, with a nonalcoholic steatohepatitis (NASH) activity score ≥4 (scale 0–8) and fibrosis stage of F2 or higher (scale 0–4), is defined as at-risk MASH given its higher risk for liver-related adverse outcomes (48). At-risk MASH patients are of interest as a population targeted for pharmaceutical treatment (46).

### 5.1. Epidemiology and Natural History

The overall epidemiology of MASLD is challenging to define due to a prolonged asymptomatic phase and variable methodology used for diagnosis in epidemiologic studies. Global prevalence is estimated at 25% and US-specific prevalence at 30% (49, 50), and this is anticipated to increase in parallel to the growing global prevalence of metabolic syndrome, diabetes, and obesity (51). Among those with MASLD, estimates of MASH range from 14% (in a prospective biopsy study) to 20% (52, 53). Prevalence of MASH as the etiology of end-stage liver disease has risen steadily over the past decade, now responsible for approximately 30% of liver transplants in the United States (54).

Mortality among persons with MASLD is primarily driven by cardiovascular disease, with lesser contributions from diabetes, extrahepatic cancers, and liver-related causes. Even in the absence of fibrosis, MASLD is associated with an increased risk for overall mortality (55). However, among those with MASLD and advanced fibrosis and cirrhosis, liver-related causes are the predominant causes of mortality (55–57). An increasing stage of fibrosis is associated with a higher risk of liver-related events or liver-related mortality (57). Hepatocellular carcinoma (HCC) can develop as a consequence of advanced fibrosis but is well recognized to develop even in the absence of advanced fibrosis in persons with MASH (57).

Factors linked with the development of MASH and fibrosis progression are numerous, including other metabolic comorbidities, genetic profile, and environmental factors. A paired biopsy study found that 30% of patients with MASLD had disease progression over a median of 3.5 years between biopsies, with higher rates of progression seen when fibrosis was initially present. In those without fibrosis on initial biopsy, about 5% developed fibrosis on their second biopsy (58). The presence of MASH is associated with a rate of fibrosis progression of one stage per 7 years, while simple steatosis is associated with a rate of one stage per 14 years (59). Greater histologic disease activity is also associated with a higher risk for fibrosis progression (48, 60). Despite these low rates of progression, it is estimated that approximately 30% of persons with MASLD will eventually develop advanced fibrosis (stages F3 or F4) (57).

By definition, persons with MASLD have at least one cardiometabolic risk, and many individuals have several comorbidities of the metabolic syndrome. Central adiposity, measured by waist circumference, is associated with risk for MASLD progression (61). Rates of MASLD, MASH, and advanced fibrosis increase with each BMI category (62, 63). In contrast to MASLD in those with comorbid obesity, the phenotype of lean MASLD is less prevalent and associated with fewer cardiometabolic comorbidities and more favorable biochemical profiles, but a greater degree of histologic fibrosis, incidence of chronic kidney disease, liver-related events, and overall mortality (64). T2DM is the comorbidity most strongly associated with MASLD and fibrosis progression (46). Up to 70% of persons with T2DM have hepatic steatosis on imaging and 20% have evidence of increased liver stiffness. The relationship between T2DM and MASLD is bidirectional, with insulin resistance present in patients with MASLD even in the absence of overt T2DM and the presence of MASLD associated with a twofold risk of incident T2DM development (65). Hypertension is a common comorbidity with MASLD, with an incidence of 14.5 cases per 100 person-years in MASLD-related cirrhosis (57). Lipid abnormalities and the risk for coronary artery disease are higher in patients with MASLD. Obstructive sleep apnea is associated with MASLD, with its presence associated with worse histological features and its pathologic intermittent hypoxia linked to insulin resistance (66).

In addition to cardiometabolic disorders, other endocrine disorders of the hypothalamic axis are frequently comorbid with MASLD. Thyroid hormones act on thyroid hormone receptor β (THR-β) in the liver, which leads to reduced intrahepatic TG content (46, 67). The exact relationship between thyroid disorders and risk for MASLD is currently uncertain (68). Persons with MASLD have lower levels of growth hormone, with these levels correlating with disease stage (69). Treatment of growth hormone deficiency with growth hormone receptor agonists or insulin-like growth factor 1 (IGF-1) improves comorbid MASLD (70) and treating patients with MASLD (without growth hormone deficiency) with recombinant growth hormone improves hepatic steatosis (71). Testosterone levels are associated with intrahepatic TG content and are lower in males with MASLD and elevated in females with MASLD (72). Testosterone supplementation has been shown to decrease hepatic steatosis in males (68). Population-based cohort studies suggest that postmenopausal women have a higher prevalence of MASLD, with earlier age of menopause associated with greater risk for fibrosis development (73). Polycystic ovarian syndrome, with its associated hyperinsulinemia and excessive androgen production, is associated with greater prevalence of MASLD and greater severity of steatohepatitis (74).

MASLD has significant heritability (Table 2), with significant hepatic steatosis associated with approximately 30% heritability and the risk of advanced liver fibrosis being 12.5-fold higher in first-degree relatives of persons with MASLD cirrhosis. The *PNPLA3* genetic variant I148M is responsible for the largest proportion of genetic predisposition to MASLD, with risk increased across the entire spectrum of MASLD from steatosis to cirrhosis, including higher risks for HCC and liver-related mortality (75).

### 5.2. Screening and Diagnosis of MASLD

Hepatic steatosis is often diagnosed via abdominal imaging obtained for abnormal liver enzyme levels or other minor abdominal complaints. While the gold standard for diagnosis of steatohepatitis is liver biopsy, noninvasive tests (NITs) play an important role in population screening and risk stratification.

Quantification of hepatic steatosis can be achieved with vibration-controlled transient elastography (VCTE), using controlled attenuation parameter (CAP), or with magnetic resonance elastography (MRE), using magnetic resonance imaging proton density fat fraction (MRI-PDFF). MRI-PDFF is more accurate at all degrees of steatosis. Shear-wave ultrasound is an emerging imaging technique that may be more accurate at quantifying hepatic steatosis than VCTE (76). Liver stiffness measured by elastography is an important noninvasive surrogate of fibrosis. VCTE has established validated cutoffs for prediction of early fibrosis versus advanced fibrosis (77) but is less accurate in obesity. Liver stiffness derived by MRE is more accurate than VCTE-derived liver stiffness, is not affected by obesity, and is predictive of future clinical progression, decompensations, and mortality (76, 78).

Guidelines recommend screening for MASLD and the presence of clinically significant fibrosis in patients with T2DM, medically complicated obesity, or those with a family history of cirrhosis due to MASLD (46). Screening algorithms recommend validated NITs such as the FIB-4 index or enhanced liver fibrosis test (ELF), often used sequentially to optimize sensitivity. Liver biopsy is recommended only when testing is conflicting or there is a need to evaluate for alternate diagnoses (46). Other blood biomarker–based NITs are in various stages of development for diagnosis and stratification of various stages of MASLD, including NIS4, OWLiver, PROC3, Fibrometer VCTE, FAST, AGILE, and ADAPT (79).

### 5.3. Dietary and Other Lifestyle Interventions in Persons with MASLD

**Figure 3:**
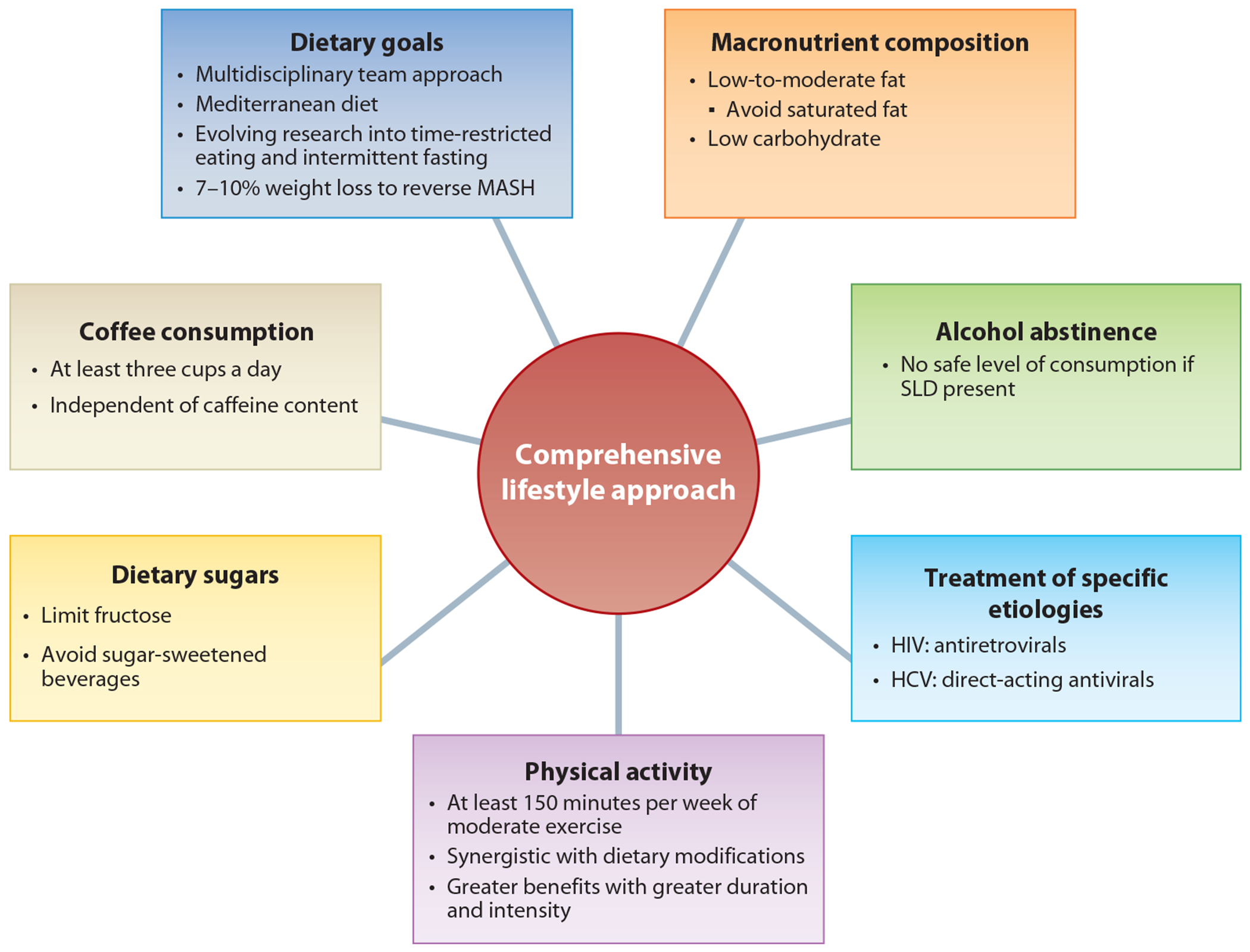
Components of lifestyle management for steatotic liver disease. Lifestyle modification forms the backbone of treatment of steatotic liver disease. Lifestyle modification broadly refers to modifications in dietary patterns, dietary composition, and physical activity levels. As effective and approved therapies for MASLD are currently limited, lifestyle modification is the main treatment option for this disease. Abbreviations: HCV, hepatitis C virus; MASH, metabolic dysfunction-associated steatohepatitis; MASLD, metabolic dysfunction–associated liver disease; SLD, steatotic liver disease.

Lifestyle modifications including dietary modifications, physical activity, and weight loss are the cornerstone of management in MASLD (Figure 3). Weight loss of 3–5% of total body weight improves hepatic steatosis and weight loss of at least 10% of total body weight reverses steatohepatitis and improves histologic fibrosis (80). Usual care is associated with only 33% achieving their targeted weight loss of 5% over three years and the majority eventually returning to their baseline weight (81). Guidelines recommend a multidisciplinary approach to weight loss that acknowledges the need for psychological support, dieticians, and nutritionists, and supporting the financial challenges of weight loss (46).

Higher dietary quality, defined by a high intake of fruits, vegetables, leafy greens, beans, and whole grains and a limited intake of refined grains, added sugars, and saturated fats, is associated with a lower risk for MASLD (82). Dietary fructose intake, in particular, is associated with increased risk of MASLD, MASH, and fibrosis progression, independent of caloric intake (83). Coffee consumption of at least three cups per day, independent of caffeine content, is associated with lower risk of MASLD (84). Physical activity of at least 150 minutes per week of moderate exercise improves steatosis, with further increases in physical activity duration and intensity above this threshold associated with greater improvements. More intensive exercise may be needed to reverse MASH. Dietary modifications and physical activity have a synergistic effect (85). High-intensity interval training has been shown to improve exercise tolerance as well as insulin resistance in a trial of patients with biopsy-proven MASH (86).

Beyond dietary composition, there is particular interest in dietary patterns such as the Mediterranean diet, intermittent fasting, and varying intensity of caloric restriction. Strict caloric restriction diets have poor patient adherence, prompting the search for better-tolerated alternatives (87). The ketogenic diet improves hepatic steatosis but is also subject to poor patient adherence (87). The Mediterranean diet, without strict caloric restriction, improved hepatic steatosis and metabolic parameters in a 12-week randomized controlled trial, whereas a comparator low-fat diet also improved hepatic steatosis with lower dietary adherence and no improvement in metabolic blood markers (88). The Mediterranean diet also reduces liver stiffness (89). The benefits of the Mediterranean diet are believed to derive from its high amounts of monounsaturated FAs and PUFAs and its balanced omega-6 to omega-3 PUFA ratio (87). Time-restricted fasting, where daily food intake is restricted to a certain window of time, and intermittent fasting, with periods of fasting up to 1–2 days per week, are dietary patterns that shift metabolic energy utilization from glycogenolysis to glucose utilization of FAs and stored adipose tissue. Use of these diets in MASLD have shown improvements in body weight, hepatic steatosis, and metabolic blood parameters (90). Time-restricted fasting studies have supported weight loss and improvement in hepatic steatosis, but they are largely limited to observational studies (91, 92). Looking to the future, with increasing use of genetics and other biomarkers, more personalized dietary and exercise prescriptions may be anticipated to achieve individual success with dietary and exercise changes.

### 5.4. Available Therapies for Treatment of MASH

Resmetirom is the first therapy for at risk MASH, approved by the US Food and Drug Administration (FDA) in March 2024 (93). Other medications, including vitamin E, pioglitazone, glucagon-like peptide-1 receptor (GLP-1r) agonists, and sodium-dependent glucose cotransporter 2 inhibitors (SGLT2i) have been studied in MASLD/MASH but lack FDA approval for this indication.

#### 5.4.1. THR-β agonists.

Resmetirom is a THR-β agonist with a 28-fold greater selectivity for THR-β than T3 and improves intrahepatic TG content and other histologic features of MASLD (94). Thyroid hormone mediates effects on hepatic glucose and lipid metabolism, THR-β mRNA levels in the liver have been shown to inversely correlate with NASH activity score (NAS), (95) and THR-β is also implicated in the fibrogenesis pathway of hepatic stellate cells (96). In the MAESTRO-NASH trial, a randomized placebo-controlled trial of resmetirom (80 mg or 100 mg) versus placebo in patients with MASH and F1–F3 fibrosis, resmetirom demonstrated superiority at 52 weeks in the primary endpoints of resolution of MASH without worsening of fibrosis (26% and 30% for resmetirom versus 10% in placebo group) and improvement in fibrosis of at least one stage without NAS worsening (24% and 26% for resmetirom versus 14% for placebo). Resmetirom had neutral effects on weight and cardiometabolic parameters, but it requires further studies to determine its effects on the thyroid hormone axis (97).

#### 5.4.2. Vitamin E.

As intrahepatic FA accumulation leads to increased oxidative stress, vitamin E has been investigated as a therapy for MASH due to its antioxidant mechanism. In a large multicenter, placebo-controlled trial (PIVENS) of adults with MASH and without diabetes, treatment with vitamin E 800 IU daily for 96 weeks was associated with significantly greater improvement in steatohepatitis, reduction in hepatic steatosis, reduced lobular inflammation, and reduced aspartate aminotransferase (AST) and alanine aminotransferase (ALT) levels compared to placebo. Despite these findings, vitamin E treatment did not improve fibrosis (98). Retrospective analysis of patients with MASH and advanced fibrosis treated with vitamin E demonstrated greater transplant free survival and lower rates of hepatic decompensations than matched controls, independent of T2DM (99). Guidelines support the use of vitamin E 800 IU daily in carefully selected patients with MASH without diabetes (46). Potential risks of treatment include increased risk of both hemorrhage and prostate cancer, although prospective studies of risk in MASH patients are lacking.

#### 5.4.3. Thiazolidinediones.

Thiazolidinediones have also been studied for the treatment of MASLD, given their activity on the PPARγ receptor upstream of pathways implicated in development of hepatic steatosis. In the PIVENS trial, pioglitazone treatment was not associated with improvements in steatohepatitis or fibrosis but was associated with improvement in serum AST and ALT levels and histologic lobular inflammation (98). An 18-month trial of pioglitazone plus a hypocaloric diet in biopsy-proven MASH and prediabetes or T2DM demonstrated MASH resolution and improved fibrosis in pioglitazone-treated patients compared to placebo (100). Meta-analysis suggests that pioglitazone treatment achieves improvements in fibrosis (101). Despite this, the side effect of weight gain (approximately 2.5 kg per patient) (100) has limited pioglitazone’s clinical utility, but a combination of pioglitazone and SGLT2 agonists shows promise in overcoming weight gain and achieving metabolic and MASH treatment benefits (102).

#### 5.4.4. GLP-1r agonists.

GLP-1r agonists have drawn significant interest for the treatment of MASH given their dramatic effects on T2DM treatment and weight loss. A randomized, placebo-controlled, phase II trial of injectable semaglutide demonstrated improvement in steatohepatitis without significant improvement in fibrosis stage and 13% total body weight loss in the treatment group (103). Another randomized, placebo-controlled trial of weekly semaglutide in patients with MASH and cirrhosis did not demonstrate improvement in histologic steatohepatitis or fibrosis (104). Tirzepatide is a dual glucose-dependent insulinotropic polypeptide (GIP) and GLP-1r agonist that has demonstrated up to 20% body weight loss and improvement in noninvasive hepatic steatosis (105, 106). There are currently no published studies with tirzepatide utilizing histologic MASH end points. Cotadutide, a dual GLP-1r and glucagon agonist, is under development for T2DM and has demonstrated improvement in liver enzymes and noninvasive assessment of hepatic steatosis in a phase II trial (107).

#### 5.4.5. SGLT2i.

SGLT2i have also generated interest in treatment of MASH given their significant effects on T2DM glucose control and modest weight loss. Small studies report improvement in hepatic steatosis (108). Large epidemiologic cohort studies suggest that SGLT2i agents are associated with improvement in MASLD as assessed by NITs (109).

### 5.5. Drugs in Development: Novel Drug Targets

**Figure 4:**
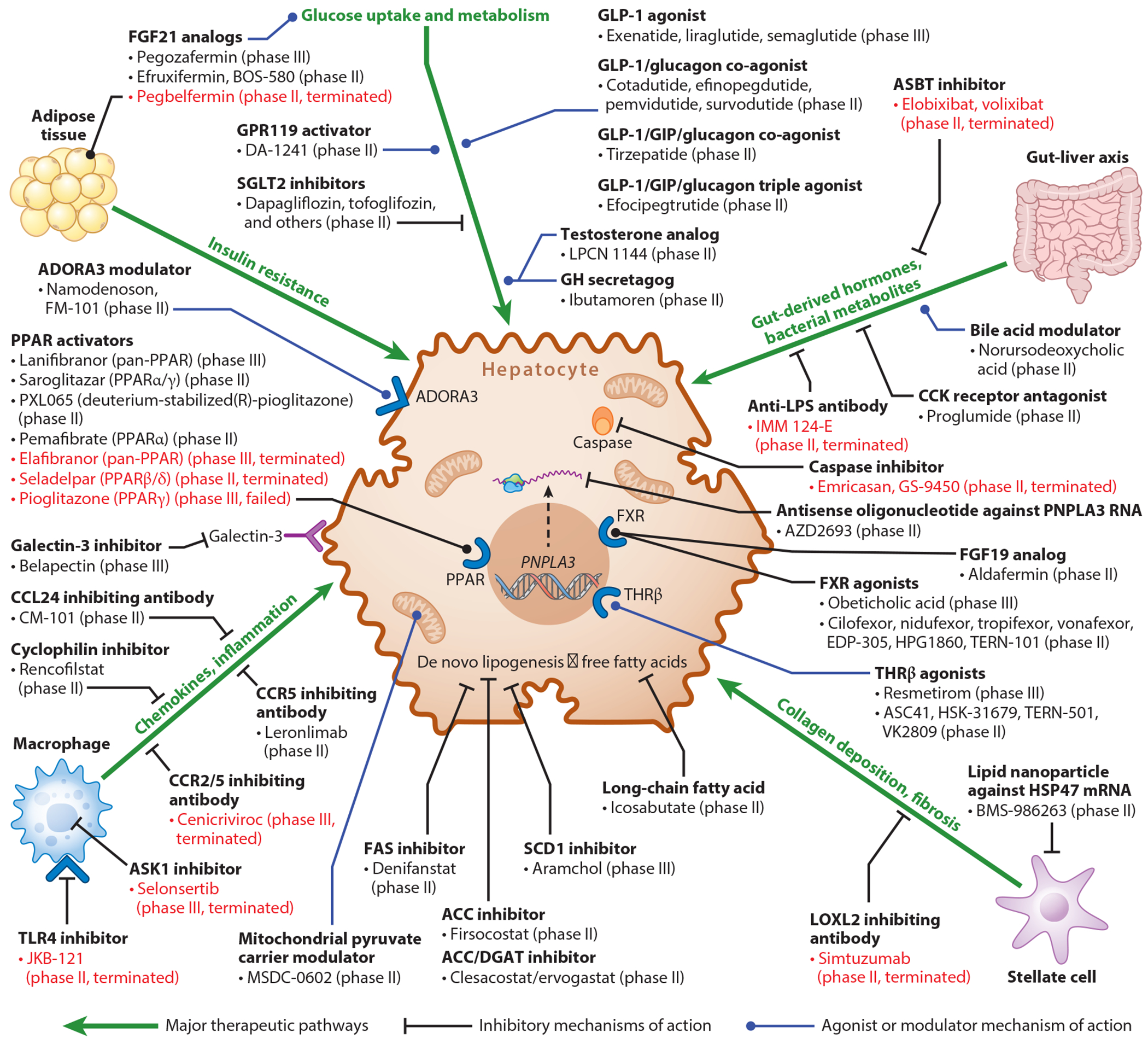
Therapeutic targets in MASLD drug development. There are multiple therapeutic targets currently in human clinical trials as well as drugs representative of these targets and currently in preclinical development. This figure is limited to products with published evidence of phase II human clinical trials or documented enrollment in phase II clinical trials and omits targets of products in preclinical development. Drugs with clearly documented termination of further trial development are depicted in red. Abbreviations: ACC, acetyl-CoA carboxylase; ADORA3, adenosine A3 receptor; ASBT, apical sodium-dependent bile acid transporter; ASK1, apoptosis signal-regulating kinase 1; CCK, cholecystokinin; CCL, chemokine (C-C motif) ligand; CCR, C-C chemokine receptor; DGAT, diglyceride acyltransferase; FAS, fatty acid synthase; FGF, fibroblast growth factor; FXR, farnesoid X receptor; GH, growth hormone; GIP, glucose-dependent insulinotropic polypeptide; GLP-1, glucagon-like peptide-1; GPR119, G protein–coupled receptor 119; HSP, heat shock protein; LOXL, lysyl oxidase homolog; LPS, lipopolysaccharide; MASLD, metabolic dysfunction–associated liver disease; mRNA, messenger RNA; *PNPLA3*, patatin-like phospholipase domain-containing protein 3; PPAR, peroxisome proliferator-activated receptor; SCD, stearoyl-CoA desaturase; SGLT2, sodium-glucose cotransporter 2; THRβ, thyroid hormone receptor beta; TLR4, Toll-like receptor 4. Figure adapted from image created in BioRender; Dukewich M. 2024. https://BioRender.com/h66p182.

Given the complex interplay of metabolic and intrahepatic factors contributing to the pathogenesis of MASLD, there are many therapeutic targets of interest to drug development, including pathways of inflammation, pathways of intrahepatic fibrosis, and metabolic pathways of lipid processing (96). Given the association of at-risk MASH with disease progression and development of advanced liver disease, the population of focus for new drug development is patients with at-risk MASH (F2 or higher) and not all persons with MASLD. These agents and the pathways targeted are shown in Figure 4.

Nuclear receptors are responsible for modulating glucose, lipid, and cholesterol metabolism. Nuclear receptors that are of interest in MASH include PPARα, PPARβ/δ, and PPARγ, FXR, and THR-β. PPARα is found in the liver and other organs with high oxidative metabolic activity and plays a role in decreased lipogenesis and increased glycogenolysis. PPARα agonists have not been shown to be beneficial in MASH (110, 111). PPARβ/δ is primarily expressed in liver cells and reduces insulin resistance and activation of inflammatory Kupffer cells. Seladelpar, a selective agonist of PPARβ/δ, had a phase II trial terminated early due to safety concerns (110). PPARγ is primarily expressed in adipose tissue but is upregulated in the MASLD liver and is responsible for stimulating FFA uptake and regulating pathways of fibrogenesis in hepatic stellate cells. Lanifibranor, a PPARγ agonist, has demonstrated improvement in MASH in a phase II clinical trial of participants with MASH without cirrhosis (112). Saroglitazar, a PPARα/γ agonist, has demonstrated improvement in metabolic risk parameters and noninvasive measures of hepatic steatosis in a phase II study in participants with MASH without cirrhosis (113).

FXR is primarily responsible for bile acid synthesis. FXR activation has been shown to reduce intrahepatic steatosis. Tropifexor, cilofexor, and obeticholic acid are FXR agonists that have been studied in MASH (114–116). A phase III trial of obeticholic acid in biopsy-proven at-risk MASH demonstrated improvement in noninvasive markers of fibrosis, as well as histologic fibrosis, but no resolution in MASH, but obeticholic acid was not approved due to safety concerns (116). Fibroblast growth factor 19 (FGF19) is secreted by small intestinal cells following food intake and regulates glucose uptake, inhibits gluconeogenesis, and plays a role in bile acid homeostasis. Aldafermin is an analog of FGF19, and its use in a phase II, placebo-controlled trial improved NITs of fibrosis in patients with MASH-related cirrhosis but demonstrated no improvement in histologic endpoints in patients with at-risk MASH (117, 118).

Hormones secreted by hepatocytes, termed hepatokines, include fibroblast growth factor 21 (FGF21), which regulates metabolic pathways in adipose tissue and mediates hepatic steatosis and fibrosis development. Elevated serum levels of FGF21 are correlated with progressive stages of MASLD fibrosis, suggesting the development of hepatic resistance to FGF21 (96). FGF21 analogs studied in MASH include efruxifermin, pegozafermin, and pegbelfermin. Efruxifermin and pegozafermin have shown encouraging results in phase II studies of patients with MASH and F2–F3 fibrosis (119, 120). Pegbelfermin was not associated with improvement in fibrosis stage in patients with MASH and F3 fibrosis or compensated cirrhosis (121, 122).

Drug candidates targeting lipogenesis such as inhibitors of acetyl-CoA carboxylase alpha (ACACA) and fatty acid synthase (FASN), such as firsocostat, clesacostat, and denifanstat, and inhibitors of SCD such as aramchol, are in earlier stages of development (96). Firsocostat, in combination with cilofexor, has demonstrated improvement in histologic NAS in a phase II trial of patients with MASH and compensated cirrhosis (123). Structurally engineered long-chain FAs, such as icosabutate, reduce inflammation through reduction of oxidized intermediates in lipid metabolism (96, 124).

Targeting the inflammatory pathways associated with MASH has been of great interest but has not yielded impressive results to date. The ASK1 inhibitor selonsertib did not improve the histologic endpoint of fibrosis in phase III trials of MASH with F3–F4 fibrosis (125). The CC-chemokine receptor (CCR2^+^) inhibitor, cenicriviroc, which is used clinically as an HIV therapy, failed to achieve MASH resolution or fibrosis stage improvement in MASH and F2–F3 fibrosis (126). Belapectin, an inhibitor of the cytokine galectin 3, failed to show improvement in the histologic end point in a phase II trial of patients with MASH and compensated cirrhosis (96, 127).

An exciting area of therapeutics is the use of genetic modification approaches targeting *PNPLA3* risk variants via RNA interference. Targeting downstream metabolic pathways (particularly *HSD17B13*) with small molecules is under development and enrolling in early phase clinical trials targeting patients with MASH and F2–F3 fibrosis. These targets may allow prevention of MASLD development through restoring normal activity in pathways of lipid metabolism and mediating progression of hepatic fibrosis (96).

## 6. ALCOHOL-ASSOCIATED STEATOTIC LIVER DISEASES

Hepatic steatosis is a frequent finding with chronic alcohol consumption or binge alcohol use. The threshold amount of alcohol that results in hepatic steatosis and the more progressive forms of liver injury vary by individuals. Chronic alcohol use perturbs fat metabolism in several ways. Normally, alcohol is metabolized via oxidative and nonoxidative pathways, with the oxidative pathways involving alcohol dehydrogenases and microsomal cytochrome P450 enzymes, accounting for the majority of metabolism. Alcohol dehydrogenases lead to production of acetaldehyde and acetate utilizes NAD^+^ as a cofactor and produces NADH, thereby reducing the NAD^+^/NADH ratio, which promotes fat accumulation in hepatocytes by reducing FA oxidation and enhancing FA synthesis. Alcohol interferes with mitochondrial β-oxidation, through potential functional deficiencies in critical cofactors for β-oxidation such as carnitine. Alcohol inhibits AMPK, which in turn inhibits PPARα and results in an inhibition of FA oxidation. Alcohol also enhances the activation of transcription factors such SREBF1/SREBP1c, which in turn stimulates the expression of genes involved in FA synthesis. Alcohol also inhibits the secretion of FAs via VLDLs from the liver.

The progression from hepatic steatosis to steatohepatitis is the consequence of an adaptive response to the neoantigens formed when aldehyde adducts with cellular proteins and DNA and leads to generation of ROS. CYP2E1 is an inducible enzyme, with intracellular levels increasing with continuous alcohol consumption, and this in turn increases the generation of ROS and cellular oxidant stress. These changes trigger pathways, such as the UPR or the ER stress response, which if overwhelmed, lead to engagement of cell death programs (as discussed above). Injured hepatocytes release DAMPs, cytokines, and chemokines, which activate and recruit innate immune cells such as macrophages and neutrophils, driving an inflammatory and fibrogenic response. Gut and adipose dysfunction caused by alcohol contributes to inflammation and stellate cell activation, setting the stage for progressive fibrosis and cirrhosis.

Liver-gut and liver-adipose cross talk are important contributors to hepatic steatosis, inflammation, and fibrosis. Alcohol disrupts the intestinal permeability and alters microbial composition and metabolism. Alcohol-induced disruption of intestinal tight junctions coupled with microbial dysbiosis results in toxic metabolites, microbes, and associated PAMPs reaching the liver via the enterohepatic circulation and contribute to liver injury and inflammation. Alcohol also disrupts bile acid homeostasis, with increased bile acid synthesis and dysregulated bile acid synthesis, as well as lipid and glucose metabolism (via FXR signaling and other pathways). Chronic alcohol administration affects adipose metabolism and immune function. Adipose lipolytic activity is increased under chronic alcohol use, leading to increased delivery of FAs to the liver and increasing steatosis. Alcohol also increases inflammatory responses in adipose tissue, contributing to oxidative stress that leads to adipocyte cell death and further impairment of adipose metabolic function. Finally, chronic and excessive alcohol use affects the immune system and immune cells directly, potentially influencing liver injury and fibrosis progression.

### 6.1. Alcohol-Associated Liver Disease: Clinical Spectrum

ALD is a spectrum from steatosis to steatohepatitis to alcohol-associated cirrhosis, and with a severe, acute clinical presentation of alcohol-associated hepatitis (128). Women are more susceptible to the liver effects of alcohol than men, developing SLD and ALD at lower doses of alcohol and over a shorter duration of time (129). The reasons for this sex difference are not completely known but include lower total body water content and lower gastric alcohol dehydrogenase activity (130). Genetic factors, including PNPLA3 and TM6SF2 genotypes among others, epigenetic factors, and environmental factors contribute to the interindividual variation in clinical phenotype. While hepatic steatosis is frequent with chronic alcohol use [>20 g (females) and >40 g (males) per day] or binge drinking and has been associated with the development of hepatitis steatosis, only about 20% of these individuals progress to steatohepatitis and progressive fibrosis. However, hepatic steatosis predicts negative long-term consequences. In a Danish cohort of 446 patients with a history of excessive alcohol intake followed for a median of 70 months, 72% had SLD, and those with SLD had a significantly higher risk of hepatic decompensation and death than those without SLD (131).

Importantly, metabolic risks and alcohol frequently coexist, and recent nomenclature changes for SLD emphasize this (Table 1). For those with metabolic risks and average daily alcohol intake of <20/40 g for women and men, steatosis is likely due to MASLD, whereas those with daily alcohol intake of >50/60 g for women and men, steatosis is likely due mostly to ALD. A new term, MetALD, delineates persons with at least one cardiometabolic risk factor and a daily intake of 20–50 g of alcohol for females and 30–60 g daily for males (46). In a US population-based study of 7,367 adults with steatosis defined by VCTE for CAP ≥248 dB/m and alcohol use measured in the prior year, the overall prevalence of SLD was 34%, with 32% having MASLD, 2% MetALD, and 0.7% ALD (132). In a UK Biobank study where MRI-proton-density-fat-fraction ≥5% was used to define SLD, among 40,189 participants, 27% had SLD. The vast majority (91%) had MASLD, whereas 8% had MetALD and 1% ALD (1). The important convergence of metabolic derangement and alcohol in promoting hepatic steatosis and subsequent liver injury, inflammation, and fibrosis is highlighted by the nomenclature change.

The alcohol thresholds that increase risk for liver-related outcomes among those with concurrent metabolic risks are incompletely known. Using the population-based NHANES cohort of 2,834 persons with hepatic steatosis, of whom 20.8% had FIB-4 values >1.3 (reflecting a group at risk for fibrotic liver disease) and with 66,299 person-years of follow-up from baseline, a linear relationship was present between daily alcohol consumption (any amount) and mortality risk. Importantly, even in the low-risk group (FIB-4 <1.3), a threshold for increased mortality was evident at 7.4 g of alcohol per day (equivalent to half of a standard drink) (133). In another study using the NHANES cohort but limited to those with metabolic steatotic liver disease, all-cause mortality in males increased linearly with weekly alcohol consumption. However, for females the risk of all cause mortality remained relatively stable until consumption reached two drinks per week, after which it rapidly increased with each additional drink consumed to a mortality rate higher than in males (134). Aligned with the uncertainty in the evidence, current guidelines indicate there is no safe level of alcohol use among persons with evidence of liver disease (128).

### 6.2. Alcohol-Associated Liver Disease: Therapies

The cornerstone of treatment in ALD is the achievement of alcohol abstinence and provision of nutritional support. The importance of using all available interventions to treat alcohol use disorder is recognized by experts and management guidelines for ALD (128). This includes the use of medications to reduce alcohol cravings, such as acamprosate and naltrexone. Novel therapies for ALD are primarily directed toward the distinct clinical syndrome of alcohol-associated hepatitis, which is characterized clinically by jaundice and complications of liver failure in the setting of recent heavy alcohol use. This syndrome is marked by a systemic inflammatory response and carries a high short-term mortality. Corticosteroids remain the primary therapy for those with severe alcohol-associated hepatitis, with guidelines also supporting the use of N-acetyl cysteine (as antioxidant) (128). For some patients with severe alcohol-associated hepatitis, the only effective therapy is liver transplantation (135), but this is available for the minority. Thus, there is a high unmet need for therapies in those with severe alcohol-associated hepatitis. Table 3 summarizes the classes of drugs under study, the phase of study, and results to date. Overall, results have been underwhelming. The need for therapeutic trials to address both the liver disease and alcohol use disorder has been recently emphasized.

## 7. VIRAL DISEASES AND HEPATIC STEATOSIS

### 7.1. Hepatitis C Virus

Hepatitis C virus (HCV) circulates as a lipoviral particle and enters the cell by way of several entry cofactors, including the low-density lipoprotein (LDL) receptor, heparan sulfate, CD81, and scavenger-receptor-class-B-member-1, and exploits the host cell’s lipid metabolism capacity to complete its life cycle. HCV replication occurs in a specialized ER-derived membranous web that is closely associated with LDs, which are enriched in HCV core and apoB and apoE. DGAT1, located in the ER membrane, is crucial for HCV replication, colocalizing with the core protein and facilitating viral assembly (136, 137). DGAT1 also recruits HCV NS5A to LDs, aiding in the translocation of other HCV nonstructural proteins, marking a stepwise transition of HCV interaction from the ER to the LD (136, 137). For assembly and release of viral particles, HCV utilizes the VLDL secretion process. Further, through activation of the NLRP3 inflammasome in HCV-infected cells, SREBP1c generation is increased, leading to de novo TG synthesis (138). HCV promotes LDL receptor expression and increased uptake of cholesterol, while inhibiting VLDL secretion and promoting LD formation (138). Additionally, HCV induces lipogenesis by downregulating hepatocyte nuclear factor 4 alpha, which controls the expression of microRNA mir-122, thereby inhibiting NF-κB-inducing kinase and promoting lipogenesis (139). This dysregulation of lipid metabolism not only leads to hepatic steatosis but also contributes to the direct cytopathic effects of the virus and renders the hepatocytes more susceptible to ER stress and subsequent downstream consequences.

Hepatic steatosis, hypocholesterolemia, and insulin resistance are frequent findings among HCV-infected persons. The association of HCV and steatosis differs by genotype, with genotype 3 linked more directly with viral-associated steatosis, whereas non-3 genotypes are associated with cardiometabolic risks, including obesity, visceral adiposity, and insulin resistance (140). In support of the specific association of steatosis with genotype, evaluation of patients before and after achievement of viral cure showed no change in hepatic steatosis if they were HCV genotype 1, irrespective of the treatment response, whereas in those infected with HCV genotype 3, treatment response was associated with significantly reduced steatosis (141). Moreover, the hypocholesterolemia of HCV infection (any genotype) reverses upon viral clearance with increases in total and LDL cholesterol (142). Cohort and case-control studies comparing HCV-infected persons with versus without virologic cure show significant reductions in cardiovascular disease and T2DM, supporting the causal link between HCV and steatosis, with reversal of steatosis-related comorbidities with achievement of virologic cure (143).

### 7.2. HIV-Related Hepatic Steatosis

Hepatic steatosis in persons living with HIV (PLWH) is the consequence of metabolic syndrome (as in the non-HIV population) as well as unique factors associated with HIV. Long-time HIV infection, even with viral suppression, is associated with immune activation, promoting a pattern of accelerated, premature immune aging and metabolic burdens associated with aging referred to as inflamm-aging (144). HIV infection affects the intestinal wall integrity and reduces microbiota diversity, changes resulting in chronic immune stimulation. Lipodystrophy, a metabolic complication of HIV infection and antiretroviral therapy, changes adipocyte size and function, with atrophy of subcutaneous adipose tissue and accumulation of fat in other areas, including in the liver. HIV also causes mitochondrial dysfunction (145). These effects of immune system activation, mitochondrial dysfunction, and cellular senescence facilitate liver injury and promote fibrosis.

MASLD is common in PLWH. In a meta-analysis of 24 articles published between 2009 and 2022 with 6,326 PLWH included, the global pooled prevalence of MASLD was 38%, and significant liver fibrosis was present in 13% (146). A US-based study of 342 PLWH defining SLD by CAP >263 dB/m found a prevalence of steatosis of 51%, with most due to MASLD (90%) rather than alcohol (147). In a meta-analysis of 13 studies of liver biopsies in PLWH and steatosis, the prevalence of MASH was 49% and significant liver fibrosis (≥F2 on histology) was 23% (148). Whether the natural history of MASLD/MASH is accelerated in PLWH compared to those without HIV infection is unclear. Using the placebo arm of a clinical trial with paired biopsies, 38% showed hepatic fibrosis progression over 12 months, with higher visceral fat at baseline identified as a risk factor (149). In contrast, a longitudinal study for 41 PLWH and 28 controls followed by 16 years with liver fibrosis estimated by elastography found no difference in fibrosis progression (150). Finally, in a propensity-matched cohort of US military veterans with MASLD, 920 with concurrent HIV infection and 920 without, there were no significant differences in risks of cirrhosis or HCC (126).

HIV viral load is not associated with risk of MASLD or MASH (148). First-generation antiretroviral therapy, specifically protease inhibitors and non-nucleoside reverse transcriptase inhibitors, was associated with metabolic derangements including lipodystrophy and hepatic steatosis, but these drugs are generally reserved now for salvage regimens. Integrase strand transfer inhibitors combined with nucleoside reverse transcriptase inhibitors are now the preferred first-line therapy. Weight gain, increased insulin resistance, and elevated TGs are reported with the use of tenofovir alafenamide (versus tenofovir disoproxil fumarate) combined with integrase inhibitors, but whether the specific integrase inhibitors used are an additional factor is unclear.

Current US guidelines do not recommend routine screening for hepatic steatosis in PLWH. As in persons without HIV, lifestyle measures to optimize metabolic health and reduce alcohol use remain important cornerstones of management. There is a need to consider antiretroviral drug effects on metabolic profiles in this context, although there are no guideline recommendations for HIV treatment in the context of MASLD. Studies of liver-specific therapies in PLWH are limited and have included small studies in vitamin E, aramchol, maraviroc, and tesamorelin (a growth hormone-releasing hormone analog approved for treatment of HIV lipodystrophy). Tesamorelin given subcutaneously daily for 12 months was associated with decreased steatosis on MRI-PDFF (absolute reduction compared to placebo of −4.1%, corresponding to a −37% (*p* = 0.016) relative reduction from baseline (151).

## 8. CONCLUSIONS

The liver plays a central role in lipid metabolism, and steatosis is a key manifestation of the transition from metabolic health versus unhealth. Hepatic steatosis should never be viewed as a benign finding. Steatosis identifies a state of risk for metabolic perturbations and can be associated with liver-related injury and inflammation, which can be progressive. The nomenclature for steatotic liver disease has recently been clarified and includes MASLD, MetALD, ALD, monogenic conditions including viral causes, and cryptogenic steatotic liver disease. MASLD is the most prevalent etiology of steatotic liver disease, affecting nearly one-third of the global population. Substantial progress has been made in defining the natural history of MASLD, improving noninvasive diagnostic tools, and defining therapeutic interventions, with the first therapy for at-risk MASH recently approved by the FDA. Indeed, the therapeutic pipeline for MASLD is quite robust, with multiple different drug targets. In contrast, the paucity of therapeutic trials in ALD and MetALD represents a therapeutic area of high unmet need.

## Figures and Tables

**Table 1:** Classification of steatotic liver diseases

Subtypes	Presence of cardiometabolic risk factors^a^	Alcohol use (daily average F/M)^b^	Genetic risks
**Hepatic steatosis**			
MASLD	+	<20/<30 g	+
MetALD	+	20–50/30–60 g	+
ALD	+/−	>50/>60 g	+
Viral	+/−	<20/<30 g	+/−
Other monogenic^c^	−	<20/<30 g	+/−
Cryptogenic	−	<20/<30 g	Unknown

a Risk factors must include at least one of the following: BMI, waist circumference, diabetes, hypertension, or dyslipidemia.

b One alcoholic drink equals 14 g of ethanol.

c Medication-induced, Wilson disease, lysosomal acid lipase deficiency, hypobetalipoproteinemia, celiac disease, and malnutrition.

Abbreviations: ALD, alcohol-associated liver disease; BMI, body mass index; F/M, females/males; MASLD, metabolic dysfunction–associated steatotic liver disease.

**Table 2:** Key genetic variants linked with risk of progressive/advanced steatotic liver disease

Gene	rs	Putative function	MASLD	ALD
*PNPLA3*	rs738409	Encodes a class I ER bilayer LD protein that carries triglyceride hydrolase activity, thereby impeding lipolysis; also affects hepatic stellate cells	↑	↑
*TM6SF2*	rs58542926	Regulates fat metabolism influencing triglyceride secretion and hepatic lipid droplet content; impedes VLDL assembly and export	↑	↑
*SAMM50*	rs2143571	Maintains mitochondrial cristae structure and proper assembly of the mitochondrial respiratory chain complexes and FA oxidation	↑	NA
*SUGP1*	rs10401969	Regulates cholesterol metabolism	NA	↑
*TRIB1*	rs2980888, rs2954038	Modulates hepatic lipogenesis and glycogenesis via multiple molecular interactions	↑	
*MBOAT7*	rs641738	Acyltransferase involved in regulation of free arachidonic acid through the remodeling of phospholipids	↑	↑
*FAF2*	rs11134997	Sensor of intracellular levels of long-chain unsaturated fatty acids; also regulates SREBP1 activation and triacylglycerol synthesis from diacylglycerol	NA	↑
*GCKR*	rs70094	Regulates glucokinase by forming an inactive complex with this enzyme	↑	NA
*HSD17B*	rs72613567	Involved in lipoprotein metabolism and positive regular of lipid biosynthesis	↓	↓

Abbreviations: ↑, increased risk; ↓, decreased risk; ALD, alcohol-associated liver disease; ER, endoplasmic reticulum; FA, fatty acid; LD, lipid droplet; MASLD, metabolic dysfunction–associated liver disease; NA, not applicable/association not reported; rs, reference single nucleotide polymorphism (SNP) cluster ID; SREBP1, sterol regulatory element-binding protein 1; VLDL, very-low-density lipoprotein.

**Table 3:** Novel therapies for alcohol-associated hepatitis

Targeted mechanism	Drug	Phase of study	Outcomes
Anti-inflammatory	Pan-caspase inhibitor (emricasan)	II	Terminated
	ASK1 inhibitor (selonsertib)	II	No benefit compared to prednisolone (NCT02854631)
	IL-1 blockade (anakinra + zinc ± pentoxifylline)	II	No benefit, stopped early, recruiting
	IL-1b monoclonal Ab (canakinumab)	II	No benefit, stopped early, recruiting
Antioxidant	N-acetyl cysteine	III	Short-term mortality (28 days) benefit in combination with prednisolone
	Metadoxine	IV	A 3- and 6-month mortality benefit in combination with prednisolone
	Vitamin E	II/III	No benefit
Gut-liver modulation	Bovine colostrum	II/III	No results available
	Probiotics	II	Mixed results, mainly focused on AST/ALT improvement
	FMT	II	Safe and improved 90-day survival
	Antibiotics (amoxicillin-clavulanate)	III	No difference in 60-day mortality compared with prednisolone
Hepatic regeneration	IL-22	II	Reduced MELD and Lille scores
	GCSF	II	Mixed results on mortality benefit
Epigenetic modulation	Larsucosterol	III	90-Day mortality numerically but not statistically higher than standard of care

Abbreviations: Ab, antibody; ALT, alanine aminotransferase; AST, aspartate aminotransferase; FMT, fecal microbiota transplant; GCSF, granulocyte colony–stimulating factor; IL, interleukin; MELD, Model for End-Stage Liver Disease.
